# An alternative downstream translation start site in the non‐TIR adaptor Scimp enables selective amplification of CpG DNA responses in mouse macrophages

**DOI:** 10.1111/imcb.12540

**Published:** 2022-03-22

**Authors:** James EB Curson, Lin Luo, Liping Liu, Belinda J Burgess, Nilesh J Bokil, Adam A Wall, Tomas Brdicka, Ronan Kapetanovic, Jennifer L Stow, Matthew J Sweet

**Affiliations:** ^1^ Institute for Molecular Bioscience (IMB), IMB Centre for Inflammation and Disease Research, and Australian Infectious Diseases Research Centre The University of Queensland Brisbane QLD Australia; ^2^ Laboratory of Leukocyte Signaling Institute of Molecular Genetics of the Czech Academy of Sciences Prague Czech Republic; ^3^ Friedrich Miescher Institute for Biomedical Research Basel Switzerland

**Keywords:** Adaptor protein, alternative translation start site, CpG DNA, macrophage, protein translation, Toll‐like receptor

## Abstract

Toll‐like receptor (TLR) signaling relies on Toll/interleukin‐1 receptor homology (TIR) domain‐containing adaptor proteins that recruit downstream signaling molecules to generate tailored immune responses. In addition, the palmitoylated transmembrane adaptor protein family member Scimp acts as a non‐TIR‐containing adaptor protein in macrophages, scaffolding the Src family kinase Lyn to enable TLR phosphorylation and proinflammatory signaling responses. Here we report the existence of a smaller, naturally occurring translational variant of Scimp (Scimp TV1), which is generated through leaky scanning and translation at a downstream methionine. Scimp TV1 also scaffolds Lyn, but in contrast to full‐length Scimp, it is basally rather than lipopolysaccharide (LPS)‐inducibly phosphorylated. Macrophages from mice that selectively express Scimp TV1, but not full‐length Scimp, have impaired sustained LPS‐inducible cytokine responses. Furthermore, in granulocyte macrophage colony‐stimulating factor‐derived myeloid cells that express high levels of Scimp, selective overexpression of Scimp TV1 enhances CpG DNA‐inducible cytokine production. Unlike full‐length Scimp that localizes to the cell surface and filopodia, Scimp TV1 accumulates in intracellular compartments, particularly the Golgi. Moreover, this variant of Scimp is not inducibly phosphorylated in response to CpG DNA, suggesting that it may act via an indirect mechanism to enhance TLR9 responses. Our findings thus reveal the use of alternative translation start sites as a previously unrecognized mechanism for diversifying TLR responses in the innate immune system.

## Graphical abstract

Here we report the existence of a smaller, naturally occurring translational variant of the transmembrane adaptor protein family member Scimp (Scimp TV1), which is generated through leaky scanning and translation at a downstream methionine. Unlike full‐length Scimp that localizes to the cell surface and filopodia, Scimp TV1 accumulates in intracellular compartments, particularly the Golgi, and enhances CpG DNA‐inducible cytokine production in granulocyte macrophage colony‐stimulating factor‐derived myeloid cells. Our findings thus reveal the use of alternative translation start sites as a previously unrecognized mechanism for diversifying Toll‐like receptor responses in the innate immune system.

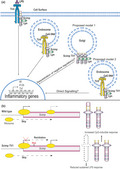

## INTRODUCTION

Macrophages are a heterogeneous population of bone marrow‐ and embryonic‐derived cells with diverse physiological functions. As innate immune sentinels, they act as first responders in the event of infection or tissue damage, coordinating host‐protective inflammatory responses.^1^ These cells express an extensive repertoire of danger‐sensing receptors, including several families of pattern recognition receptors.^2^ Pattern recognition receptors detect both exogenous pathogen‐associated molecular patterns and endogenous danger‐associated molecular patterns.^3^ The Toll‐like receptor (TLR) family of pattern recognition receptors are type I transmembrane proteins that play key roles in initiating and driving host‐protective inflammatory and antimicrobial responses.

TLRs localize to both the plasma membrane and endosomal compartments, enabling them to detect molecular signatures of different classes of pathogens (e.g. extracellular or vesicular).^4^ The most extensively studied TLR is TLR4, a receptor for the Gram‐negative bacterial cell wall component lipopolysaccharide (LPS).^5^ TLR4 signals from both the cell surface and endosomal compartments. Homodimeric TLR4 recognizes LPS at the cell surface, a process reliant on the loading of LPS onto TLR4–MD2 protein complex via cell surface or soluble CD14.^6^ The recruitment of the Toll/interleukin‐1 receptor homology (TIR) domain‐containing adaptor proteins MAL^7^ and MyD88^8^ propagates downstream signaling, with MAL facilitating the association of TLR4 and MyD88.^9^ This clustering of adaptors leads to the oligomerization of MAL and MyD88 proteins^10^ and recruitment of members of the interleukin (IL)‐1 receptor‐associated kinase (IRAK) family of serine/threonine kinases through homotypic interactions of MyD88 and IRAK death domains. This oligomerized complex represents a supramolecular organizing center referred to as the Myddosome.^11^ Structurally, the Myddosome consists of four to six MyD88, four IRAK4 and four IRAK2 molecules, acting as a signaling platform from which the IRAKs drive both mitogen‐activated protein kinase (MAPK) and nuclear factor‐κB activation.^12^ Ligand‐activated TLR4 is then internalized into endosomes,^9^ leading to the disengagement of MAL and MyD88 and the recruitment of the TIR‐domain containing adaptors TRIF and TRAM.^9^ These adaptors still promote nuclear factor‐κB signaling, but also initiate the phosphorylation and activation of IRF3.^4^ However, some endosomal TLRs, for example, TLR9 that detects unmethylated CpG‐containing DNA (CpG DNA),^13^ act independently of TRIF^14^ and signal through only the MyD88 pathway.^15^

An important determinant of TLR signaling responses is their localization, which is controlled by molecules involved in their journey to the sites where signaling is initiated. UNC93B, for example, is unique to the endosomal TLRs, facilitating both their transport from the endoplasmic reticulum to the Golgi, and their final localization in target endosomes.^16^ Despite the role of UNC93B in enabling responses to multiple endosomal TLRs, there is also evidence for TLR9‐specific trafficking and signaling events. TLR9 interacts with UNC93B in the endoplasmic reticulum, enabling its loading into COPII vesicles and transport to the Golgi. From here, TLR9 is reported to translocate with UNC93B to the cell surface, where this complex interacts with adaptor protein‐2 (AP‐2) to enable TLR9 endocytosis.^16^ Endosomal TLR9 first interacts with CpG DNA in a VAMP3^+ve^, LAMP2^−ve^, PI(3,5)p_2_^+ve^ endosome where it signals through MyD88, facilitating nuclear factor‐κB‐dependent activation of inflammatory genes.^17^ TLR9 is then directed to a specialized LAMP2^+ve^ lysosome‐related organelle, a process that is reliant on adaptor protein‐3 (AP‐3). Here it recruits IRF7, subsequently driving the type I interferon response.^17^ Such studies highlight the complex and unique nature of TLR9 signaling.

The transmembrane adaptor proteins (TRAPs) are a family of membrane‐bound adaptors that relay signals from receptor complexes. The palmitoylated transmembrane adaptor proteins (pTRAPs) are a subset of TRAPs with important roles in adaptive immune cell signaling.^18^ They are grouped according to shared structural features, which include a short extracellular domain, a single‐pass transmembrane domain (TMD) and a cytoplasmic tail that becomes post‐translationally modified by palmitoylation on a cysteine residue on the cytoplasmic face of the TMD. The pTRAPs themselves have no inherent enzymatic activity, but instead recruit effector proteins to elicit biological effects. Although studies of proximal TLR signaling have primarily focused on TIR‐containing adaptors, TLR TIR domains are also known to be tyrosine phosphorylated.^19^ For example, surface‐exposed Y674 of human TLR4 has been reported to be involved in TLR‐mediated activation of nuclear factor‐κB.^20^ Members of the Src family of tyrosine kinases,^21^ particularly Lyn,^20^ have been implicated in phosphorylation of the TLR4 TIR domain. We recently showed that Slp65/76 and CSK‐interacting membrane protein (Scimp), an immune‐restricted pTRAP family member that is constitutively bound to Lyn^22^ and with emerging roles in innate immunity,^23^, ^24^ acts as a noncanonical adaptor protein to promote LPS‐inducible and Lyn‐dependent TLR4 phosphorylation and downstream inflammatory responses in mouse macrophages.^25^ In so doing, Scimp selectively promotes LPS‐inducible production of certain inflammatory cytokines, particularly IL‐6 and IL‐12p40.^25^ Scimp also scaffolds the SH2 domain‐containing proteins, Slp65/76, Grb2 and Csk, in an LPS‐inducible manner, facilitating signal diversification upon TLR4 activation.^26^ We also showed that Scimp directs signaling molecule recruitment and selective cytokine production downstream of multiple TLRs.^27^

Alternative splicing provides a means of generating functionally distinct signaling molecules from the one gene.^28^ This has been demonstrated for multiple genes in the TLR signaling framework, for example, *Tlr4*, *Tollip*, *Irak1*, *Irak2* and *Irak4*.^29^ Functional consequences of differential transcript usage have been reported for several TLR signaling molecules including MyD88^30^ and IRAK2.^31^ However, comparatively little attention has been paid to functional diversification of innate immune responses through engagement of alternative translation start sites (TSSs). In eukaryotic genomes, AUG codons downstream of suboptimal TSS have enhanced conservation,^32^ and these can be used as alternative TSS as a result of “leaky scanning” by the ribosome.^33^ In some instances, proteins generated from different TSS can have distinct localizations and/or functions. For example, insulin‐degrading enzyme, which regulates cerebral amyloid beta‐peptide and plasma insulin levels *in vivo*, can initiate translation 123 nucleotides upstream from the more optimal TSS.^34^ This results in the production of a mitochondrially localized variant with unique functions. In this study, through the generation of a novel clustered regularly interspaced short palindromic repeats (CRISPR)‐modified mouse (*Mus musculus*) line, we report the existence of a translational variant of Scimp that has a distinct role in promoting TLR9 responses in macrophages.

## RESULTS

### Scimp TV1 is a truncated variant lacking the first 13 amino acids

**Figure 1:**
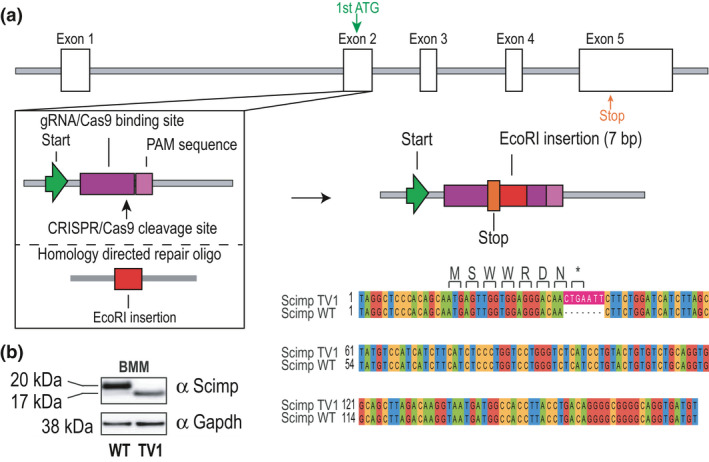
Generation of a mouse line expressing a naturally occurring truncated form of Scimp. **(a)** Scimp TV1 mice were generated using CRISPR–Cas9 insertion of a 7‐bp *Eco*RI site via homology‐directed repair. This insertion generates a premature stop codon after the seventh amino acid, as depicted by the nucleotide sequence comparison of wild‐type (WT) Scimp and Scimp TV1 (bottom right). **(b)** Total cell lysates of GM‐CSF‐BMMs from WT and Scimp TV1 mice were assessed for Scimp expression by western blot. Data are representative of at least three independent experiments and three mice per genotype. Cas9, CRISPR‐associated protein 9; CRISPR, clustered regularly interspaced short palindromic repeats; GM‐CSF‐BMMs, granulocyte macrophage colony‐stimulating factor‐derived bone marrow‐derived macrophages.

To further characterize the role of Scimp in TLR and other responses in macrophages, we used a CRISPR/CRISPR‐associated protein 9 strategy to insert a premature stop codon after the seventh amino acid in the first exon of the *Scimp* gene (Figure 1a). This was expected to generate a null *Scimp* allele; however, western blot analysis of granulocyte macrophage colony‐stimulating factor‐derived bone marrow‐derived macrophages (GM‐CSF‐BMMs) from these mice revealed a truncated protein with an approximate molecular weight of 17 kDa (Figure 1b). We noted that a faint band of the same molecular weight was also apparent in wild‐type (WT) cells (Figure 1b), with this band also being observed in previous studies on Scimp.^22^, ^25^ We therefore considered that the truncated protein may be a naturally occurring variant of Scimp.

**Figure 2:**
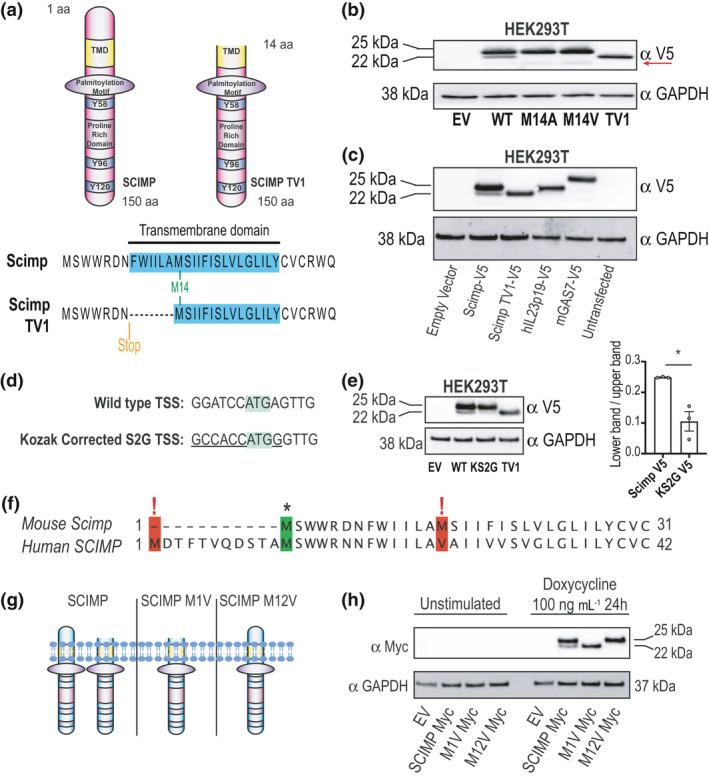
Scimp has two distant translational variants. **(a)** The inserted stop codon is predicted to result in translation from a downstream methionine residue within the transmembrane domain, leading to a truncated Scimp protein lacking the first 13 amino acids. **(b)** HEK293T cells were transfected with EV, Scimp‐V5 (WT), Scimp M14A‐V5 (M14A), Scimp M14V‐V5 (M14V) or Scimp TV1‐V5 (TV1). Total protein was collected and assessed for Scimp expression via western blot. Blots are representative of three independent experiments. The red arrow highlights a translational product that is presumed to result from the next downstream ATG in M14A and M14V. **(c)** HEK293T cells were transfected with empty vector, Scimp‐V5, Scimp TV1‐V5 or two unrelated V5‐tagged proteins (hIL23p19‐V5 and mGas7‐V5) that have downstream methionine residues within the first 15 amino acids of the first start codon. Total protein was collected and assessed for V5‐tagged protein expression via western blot. Blots are representative of three independent experiments. **(d)** A comparison of the sequence surrounding the first ATG in WT Scimp and that of a “Kozak‐corrected” version (Kozak Scimp S2G‐V5). **(e)** HEK293T cells were transfected with EV, Scimp‐V5 (WT), Kozak Scimp S2G (KS2G) and Scimp TV1‐V5 (TV1). Total protein was collected and assessed for V5‐tagged protein expression via western blot. Blots are representative of three independent experiments (left), with quantification of combined data from these experiments also displayed (right). Levels of Scimp TV1 relative to those of the upper full‐length Scimp band are displayed. Data (mean ± s.e.m., *n* = 3) are combined from three independent experiments. **(f)** The amino acid sequences of the N‐terminal regions of human SCIMP and mouse Scimp were aligned using MUSCLE. **(g)**. Predicted translational variants of human SCIMP and the isoforms that would be expected following mutation of either M1 or M12 to a valine. **(h)** THP‐1 cells were transduced with lentiviral constructs encoding EV, SCIMP‐Myc, SCIMP M1V‐Myc or SCIMP M12V‐Myc. Stably transduced THP‐1 cells were plated, differentiated with PMA for 48 h, then treated with doxycycline (100 ng mL^−1^) for 24 h to induce expression of the transduced genes. Whole‐cell lysates were collected and assessed for SCIMP protein expression via western blot. Data are representative of two independent experiments. Statistical significance in **e** was determined using an unpaired *t*‐test and GraphPad Prism 9 (*P < 0.05). EV, empty vector; PMA, phorbol‐12‐myristate‐13‐acetate; WT, wild‐type.

A second methionine residue within the predicted TMD (amino acid 14) would generate a ~17‐kDa variant lacking the first 13 amino acids if used as a downstream translation start (Figure 2a). Hereafter, we refer to this variant as Scimp translational variant 1 (Scimp TV1). To determine whether the protein product detected in macrophages from the Scimp mutant mice and the lower band observed in WT macrophages (Figure 1b) was indeed Scimp TV1, Scimp‐V5 expression constructs with the second methionine (M14) mutated to an alanine (M14A) or a valine (M14V) were created. These constructs were then expressed in HEK293T cells, alongside Scimp‐V5 and Scimp TV1‐V5, a construct lacking the first 13 amino acids of the Scimp protein. Western blot analysis revealed that the Scimp M14A‐V5 and M14V‐V5 mutants did not generate the lower band that was apparent in Scimp‐V5‐transfected cells and that corresponded to the 22‐kDa band observed in Scimp TV1‐V5‐expressing cells (Figure 2b). This confirms that Scimp TV1 is indeed generated from alternative translation from M14. Interestingly, mutation of M14 to either alanine or valine also resulted in the appearance of a faint lower molecular weight band of ~15 kDa (Figure 2b, red arrow). This is consistent with a 139‐amino acid translation product that would be generated from initiation at the next in‐frame ATG. If this ~15‐kDa band is an even smaller translational variant of Scimp, it is unlikely to be physiologically relevant since we did not detect it in either WT GM‐CSF‐BMMs (Figure 1b) or cells overexpressing WT Scimp‐V5 or Scimp TV1‐V5 (Figure 2b). However, its presence is further evidence of engagement of a downstream TSS in the Scimp messenger RNA (mRNA).

To confirm that Scimp TV1 expression was not a general artifact arising from protein overexpression in HEK293T cells, V5‐tagged proteins of similar molecular weight and with methionine residues in the first 15 amino acids of the canonical TSS (hIL23‐19‐V5 and mGas7‐V5) were expressed in HEK293T and compared with the Scimp constructs. In cells expressing either hIL23‐19‐V5 or mGas7‐V5, we observed no lower bands at molecular weights that would be expected if the downstream start codons were being used (Figure 2c). This confirms selectivity in downstream translation start usage for the Scimp mRNA. Next, we investigated molecular mechanism(s) giving rise to the alternative translation of Scimp TV1. The 5′ untranslated region immediately upstream of the first ATG, as well as the first nucleotide downstream of this start codon, does not match the optimal mammalian Kozak sequence GCCGCC(G/A)CCAUGG (Figure 2d).^35^ This led us to consider that leaky ribosome scanning may result in the production of Scimp TV1. To investigate this hypothesis, we generated a Scimp‐V5 expression construct in which the nucleotides surrounding the start codon were mutated to match the optimal Kozak sequence (Figure 2d). When this Kozak sequence‐containing Scimp construct (KS2G‐V5) was expressed in HEK293T cells, production of the Scimp TV1‐V5 protein was significantly reduced by comparison to cells expressing WT Scimp‐V5 (Figure 2e). This confirms that leaky scanning and ribosomal slippage from the first TSS in Scimp results in the production of Scimp TV1.

We next assessed whether human cells also use an alternative TSS in the human SCIMP mRNA to generate a second protein product. The second methionine (M14) used in mice to translate Scimp TV1 is substituted for valine in human SCIMP; however, the first methionine in mouse Scimp (M1) corresponds to the second methionine (M12) in human SCIMP (Figure 2f). This second methionine in human SCIMP could therefore be used as an alternative translational start site, thus enabling the generation of two SCIMP variants in human cells (Figure 2g). To interrogate this hypothesis, SCIMP‐Myc, SCIMP M1V‐Myc and SCIMP M12V‐Myc lentiviral constructs (Figure 2g) were transduced into the monocytic THP‐1 cell line to enable doxycycline‐inducible expression. As predicted, two protein products were detected in THP‐1 cells expressing WT SCIMP, and mutating M1 or M12 to valine led to the ablation of the upper and lower SCIMP isoforms, respectively (Figure 2h). We therefore conclude that leaky scanning of the Scimp TSS occurs in both mouse and human cells, giving rise to a shortened translational variant in both cases.

### Bone marrow‐derived macrophages selectively expressing Scimp TV1 display impaired IL‐12p40 production in response to sustained LPS treatment

We next assessed whether loss of full‐length Scimp and overexpression of Scimp TV1, as is apparent in Scimp TV1 mice, influences TLR‐inducible cytokines that we previously showed were Scimp dependent (IL‐6, IL‐12p40).^25^ These experiments revealed that there was no difference in the LPS‐inducible production of IL‐6, IL‐12p40 and TNF at the mRNA or protein level in colony‐stimulating factor 1 (CSF‐1)‐derived BMMs (CSF‐1‐BMMs; Supplementary figure 1a–f). Members of the pTRAP family can play compensatory roles in cell signaling, often necessitating the genetic deletion of multiple family members before a phenotype is revealed.^23^ We therefore assessed the expression of other pTRAP family members in WT *versus* Scimp TV1 CSF‐1‐BMMs. Whereas expression of *Lat1*, *Lat2* and *Lst1/A* was comparable between these cell populations, basal expression of *Pag1* was reduced in Scimp TV1 BMMs (Supplementary figure 1g–k). It is possible that our observed reduction in *Pag1* gene expression may reflect regulatory mechanisms caused by the overexpression of Scimp TV1, leading to compensatory mechanisms that permit optimal TLR responses in the absence of full‐length Scimp.

**Figure 3:**
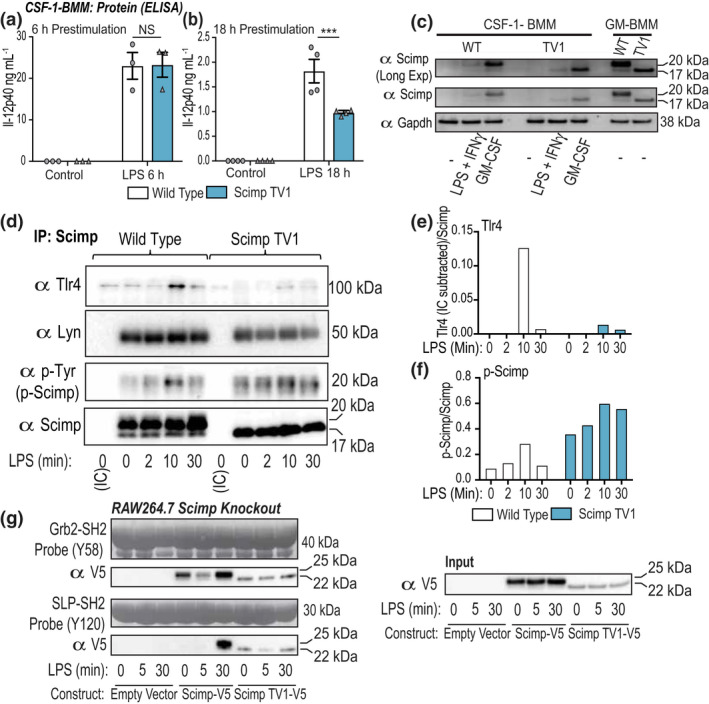
Scimp is required for sustained LPS responses and Scimp TV1 is basally, rather than LPS‐inducibly, phosphorylated. **(a, b)** BMMs from wild‐type (WT) and Scimp TV1 mice were treated with LPS (1 ng mL^−1^) for the indicated time points, before being washed of LPS and replated in fresh media for a further 24 h. Supernatants were collected and assessed for IL‐12p40 production by ELISA. Data (mean ± s.e.m., *n* = 3 or 4 mice per genotype) are combined from three or four independent experiments. **(c)** BMMs from WT and Scimp TV1 (TV1) mice were differentiated with either CSF‐1 (10 ng mL^−1^; lanes at left) or GM‐CSF (10 ng mL^−1^; lanes at right) for 6 days. CSF‐1‐BMMs were stimulated with LPS (10 ng mL^−1^) and IFNγ (5 ng mL^−1^) or GM‐CSF (10 ng mL^−1^) for 24 h. Total protein was collected and assessed for Scimp expression via western blot. Data are representative of two independent experiments collected from two mice per genotype. **(d)** BMMs from WT and Scimp TV1 mice were differentiated using CSF‐1, then primed overnight with GM‐CSF (10 ng mL^−1^). BMMs were stimulated with LPS (10 ng mL^−1^) for the indicated time points before being lysed. Collected protein was immunoprecipitated using a rabbit anti‐Scimp antibody or an isotype control (rabbit anti‐Myc tag). Immunoprecipitated samples were assessed for levels of phosphorylated Scimp (p‐Tyr) and coimmunoprecipitated TLR4 and Lyn via western blot. Data are representative of two independent experiments. **(e)** TLR4 and **(f)** phospho‐Scimp levels from **d** were quantified using Image Lab and displayed relative to the corresponding immunoprecipitated Scimp band (the IC background signal in control lanes was subtracted from the appropriate sample band intensity before quantifying). Data are representative of two independent experiments collected from two mice per genotype. **(g)** SH2 pulldown assays were used to assess LPS‐inducible phosphorylation of Y58 (Grb2‐SH2) and Y120 (SLP‐SH2) on Scimp‐V5 in RAW264.7 cells expressing Scimp‐V5 or Scimp TV1‐V5. Data are representative of three independent experiments. Statistical significance in **a** and **b** was determined using two‐way ANOVA, followed by Bonferroni’s multiple comparison test using GraphPad Prism 9 (***P < 0.001). GM‐CSF‐BMM, granulocyte macrophage colony‐stimulating factor‐derived bone marrow‐derived macrophages; IC, isotype control; IFN, interferon; LPS, lipopolysaccharide.

It was previously found that SCIMP‐dependent biological effects observed upon *SCIMP* silencing in B cells^22^ were not recapitulated when assessing functional responses in B cells from *Scimp* ^−/−^ mice.^24^ The same study showed that defects in Dectin‐1 signaling in *Scimp*‐deficient dendritic cells were only observed following sustained treatment with the Dectin‐1 agonist zymosan.^24^ To investigate whether a similar phenomenon may apply to LPS responses, we treated WT and Scimp TV1 BMMs with LPS, after which cells were washed to remove the initial stimulus and left for a further 24 h. Supernatants were collected and assessed for inflammatory cytokines at the protein level. Whereas LPS‐induced secretion of IL‐12p40 was not affected in Scimp TV1 CSF‐1‐BMMs that were pretreated with LPS for 6 h (Figure 3a), there was a significant reduction (~50%) when these cells were pretreated for 18 h with LPS (Figure 3b). These findings suggest that deletion of full‐length Scimp and/or overexpression of Scimp TV1 impair sustained cytokine production in LPS‐activated macrophages.

Given the above data suggesting that Scimp can propagate sustained LPS‐inducible inflammatory responses, we next examined whether specific inflammatory mediators enhance Scimp expression. In addition to LPS, we confirmed that GM‐CSF upregulates Scimp protein expression in CSF‐1‐BMMs (Figure 3c), as has previously been reported.^24^ The magnitude of the response was much greater than was observed for the classical macrophage‐activating combination of LPS + interferon‐γ. Moreover, GM‐CSF‐BMMs expressed much higher levels of Scimp protein in both WT and TV1 BMMs than were observed in CSF‐1‐BMMs derived from the same bone marrow cells (Figure 3c). We therefore used GM‐CSF‐BMMs to further investigate Scimp TV1 functions in a cell‐based system. In cells prestimulated with GM‐CSF that expressed high levels of the Scimp protein, LPS promoted Scimp phosphorylation and an interaction with TLR4 (Figure 3d–f), as we have previously shown in CSF‐1‐BMMs.^25^ By contrast, the interaction between Scimp TV1 and TLR4 was greatly reduced (Figure 3d, e). Furthermore, inducible Scimp TV1 phosphorylation was modest, and these cells instead displayed elevated basal Scimp TV1 phosphorylation (Figure 3d, f). Interestingly, Scimp TV1 still interacted with the Lyn kinase (Figure 3d), suggesting that it is likely to be signaling competent. Assessment of Scimp TV1 phosphorylation status by SH2 pulldown assays^26^ again confirmed that Scimp TV1 is basally, rather than LPS‐inducibly, phosphorylated (Figure 3g). Of note, the expression levels of Scimp TV1‐V5 were often much lower than that of Scimp‐V5 (Figure 3g, input: right‐hand side), hence the basal phosphorylation of Scimp TV1 observed following the pulldown is relatively high by comparison to the WT Scimp‐V5. These data suggest that Scimp TV1 is likely to have a distinct function to that of the full‐length Scimp protein. To gain further insight into possible functions of Scimp in GM‐CSF‐BMMs, we next assessed sustained LPS responses. Here we found that GM‐CSF‐BMMs continued to produce high levels of IL‐12p40 when the initial LPS stimulus was withdrawn 12 or 18 h after stimulation (Supplementary figure 2a). However, this sustained cytokine response was not diminished in Scimp TV1 GM‐CSF‐BMMs (Supplementary figure 2a), in contrast to our observations in Scimp TV1 CSF‐1‐BMMs (Figure 3b). Whereas GM‐CSF‐BMMs expressed much higher basal levels of Scimp than CSF‐1‐BMMs, LPS only upregulated the Scimp protein in the latter population (Supplementary figure 2b). These data are therefore consistent with a role for Scimp in sustaining LPS responses in CSF‐1‐BMMs, but not in GM‐CSF‐BMMs.

### CpG DNA‐induced inflammatory cytokine production is enhanced in GM‐CSF‐BMMs from Scimp TV1 mice

**Figure 4:**
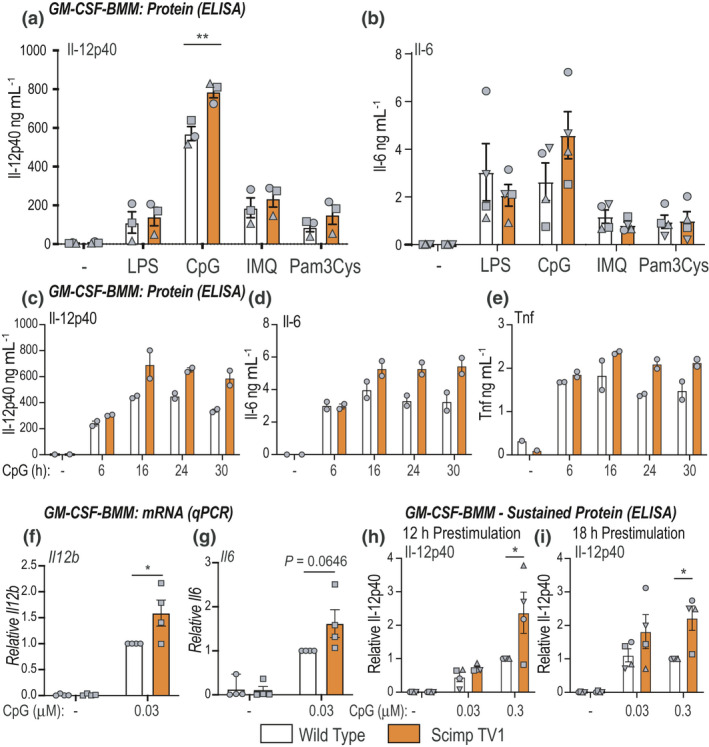
CpG DNA responses are enhanced in GM‐CSF‐derived Scimp TV1 BMMs. **(a, b)** GM‐CSF‐derived wild‐type (WT) and Scimp TV1 BMMs were stimulated with LPS (1 ng mL^−1^), CpG DNA (0.3 µM), imiquimod (25 µg mL^−1^) or Pam3Cys (15 ng mL^−1^) for 24 h. Supernatants were collected and assayed for levels of secreted IL‐12p40 and IL‐6 by ELISA. Data (mean ± s.e.m., *n* = 3 or 4 mice per genotype) are combined from three or four independent experiments (different symbols represent data from individual experiments). **(c–e)** GM‐CSF‐derived WT and Scimp TV1 BMMs were stimulated with CpG DNA (0.3 µM) for the indicated times. Supernatants were collected and assayed for IL‐12p40, IL‐6 and TNF production by ELISA. Data shown (mean ± range, *n* = 2) are technical cell culture replicates from a single experiment. Similar results were found in two independent experiments collected from two mice per genotype. **(f, g)** GM‐CSF‐BMMs from WT and Scimp TV1 mice were stimulated with CpG DNA (0.03 µM) for 4 h. Total RNA was collected and assessed for *Il12b* and *Il6* mRNA levels by RT‐qPCR. Data (mean ± s.e.m., *n* = 4 mice per genotype) are combined from four independent experiments. **(h, i)** GM‐CSF‐derived WT and Scimp TV1 BMMs were stimulated with CpG DNA (0.03 and 0.3 µM) for the indicated time points, washed and then replated in fresh media for a further 24 h. Supernatants were collected and assessed for IL‐12p40 production by ELISA. Data (mean ± s.e.m., *n* = 4 mice per genotype) are combined from four independent experiments (different symbols represent data from individual experiments). Data in **f–i** are displayed relative to CpG DNA‐stimulated WT cells. Statistical significance in **a, b** and **f–i** was determined using two‐way ANOVA, followed by Bonferroni’s multiple comparison test using GraphPad Prism 9 (*P < 0.05, **P < 0.01). GM‐CSF‐BMMs, granulocyte macrophage colony‐stimulating factor‐derived bone marrow‐derived macrophages; LPS, lipopolysaccharide; RT‐qPCR, real time‐quantitative PCR.

To assess alternative roles for Scimp TV1 in GM‐CSF‐BMMs, we examined responses to different TLR agonists in Scimp TV1 *versus* control GM‐CSF‐BMMs. Here we consistently observed elevated IL‐6 and IL‐12p40 secretion in GM‐CSF‐BMMs from Scimp TV1 mice responding to the TLR9 agonist CpG DNA, whereas responses to other TLR agonists were unaffected (Figure 4a, b). Of note, Scimp TV1 did not affect responses to the TLR7 agonist imiquimod, suggesting that it may exert some selectivity within the endosomal TLR subfamily, promoting TLR9 but not TLR7 responses. The effects on CpG DNA‐inducible cytokine release were also observed over a time course (Figure 4c–e) and at the mRNA level (Figure 4f, g). Interestingly, CpG DNA‐inducible Tnf secretion was also elevated in Scimp TV1 GM‐CSF‐BMMs (Figure 4e). Furthermore, when assessing sustained cytokine production in response to an initial CpG DNA stimulus, Scimp TV1 GM‐CSF‐BMMs show enhanced Il‐12p40 production following both a 12‐ and 18‐h CpG DNA prestimulation (Figure 4h, i, Supplementary figure 2c, d). Given that we did not observe a similar phenotype for sustained LPS responses in GM‐CSF‐BMMs (Supplementary figure 2a), these data further highlight the selectivity of the enhanced CpG DNA response in Scimp TV1 GM‐CSF‐BMMs. We note that *Tlr9* mRNA levels in GM‐CSF‐BMMs in both the basal and CpG DNA‐stimulated state were similar between WT and Scimp TV1 cells (Supplementary figure 2e, f). Similarly, we observed no significant difference in *Scimp* mRNA levels between the two GM‐CSF‐BMM populations (Supplementary figure 2g, h). These data suggest that Scimp TV1 is unlikely to enhance CpG DNA responses by upregulating TLR9 or Scimp protein expression. We conclude that this variant of Scimp most likely acts by enhancing TLR9 signaling.

### Scimp TV1 localization and CpG DNA‐inducible signaling are distinct from those of full‐length Scimp in macrophages

**Figure 5:**
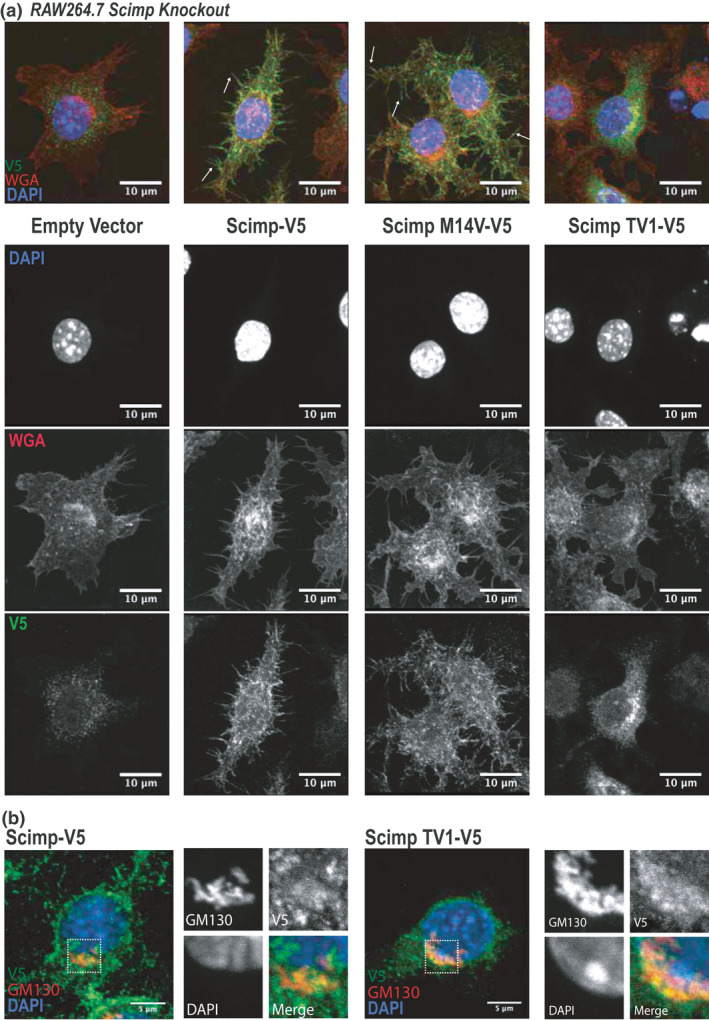
The two Scimp variants have distinct localizations. **(a)** Scimp knockout RAW264.7 cells were reconstituted with an empty vector, Scimp‐V5, Scimp M14V‐V5 or Scimp TV1‐V5. Cellular localization of V5‐tagged Scimp proteins was assessed via immunofluorescence microscopy (anti‐V5/Scimp: green; WGA: red; DAPI: blue; scale bars: 10 µm). White arrows indicate filopodia‐localized Scimp‐V5 and Scimp M14V‐V5. Similar results were observed in three independent experiments. **(b)** Scimp knockout RAW264.7 cells were reconstituted with Scimp TV1‐V5. Cellular localization of V5‐tagged Scimp alongside a Golgi apparatus‐specific protein (GM130) was assessed via immunofluorescence microscopy (anti‐V5/Scimp: green; GM130: red; DAPI: blue; scale bars: 5 µm). Similar results were observed in two independent experiments. DAPI, 4′,6‐diamidino‐2‐phenylindole; WGA, wheat germ agglutinin.

**Figure 6:**
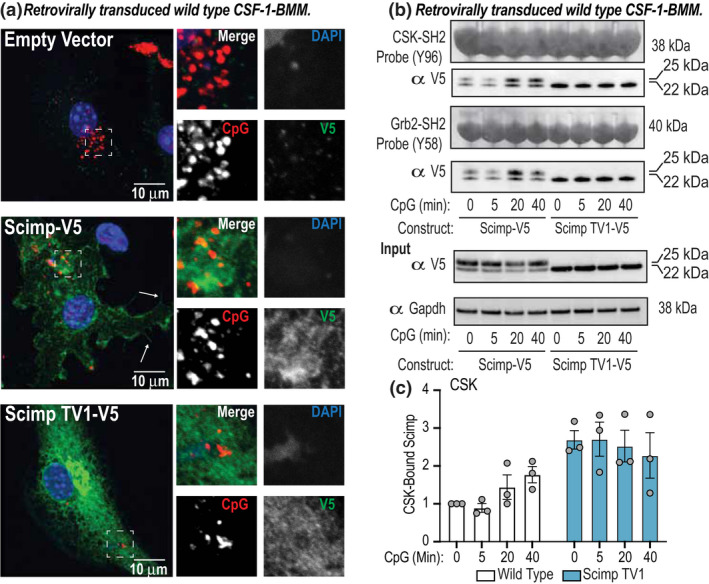
Assessment of Scimp cellular localization and phosphorylation in response to CpG DNA. **(a–c)** Wild‐type (WT) BMMs retrovirally transduced with either an empty vector, Scimp‐V5 or Scimp TV1‐V5 expression construct. Cells were treated with 0.3 µM fluorescently labeled (Alexa‐647‐conjugated) CpG DNA (CpG) for 30 min. Cellular localization of V5‐tagged Scimp proteins and CpG DNA were assessed via immunofluorescence microscopy (anti‐V5/Scimp: green; CpG‐647: red; DAPI: blue; scale bars: 10 µm). White arrows indicate filopodia‐localized Scimp‐V5. Similar results were observed in three independent experiments in which a total of three WT C57Bl/6 mice were retrovirally transduced (1 mouse per experiment). **(b)** SH2 pulldown assays were used to assess CpG DNA‐inducible phosphorylation of Y58 (Grb2‐SH2) and Y96 (CSK‐SH2) on Scimp‐V5 in CSF‐1‐BMMs expressing Scimp‐V5 or Scimp TV1‐V5. Data are representative of three independent experiments and three mice per genotype. **(c)** CSK‐bound Scimp and Scimp TV1 bands from **b** were quantified and normalized to the 0‐min control of the WT. Data (mean ± s.e.m., *n* = 3 mice per genotype) are combined from three independent experiments. BMM, bone marrow‐derived macrophages.

To determine whether Scimp TV1 has a distinct localization to full‐length Scimp that might permit a functional role in CpG DNA responses, we expressed V5 epitope‐tagged versions of WT Scimp (generating both full‐length Scimp and Scimp TV1), Scimp M14V (generating only full‐length Scimp) and Scimp TV1 (generating only full‐length Scimp TV1) in Scimp‐deficient RAW264.7 cells (Figure 5a). While Scimp‐V5 and Scimp M14V‐V5 primarily localized to filopodia at the plasma membrane as anticipated,^25^ Scimp TV1‐V5 was not observed at the cell surface (Figure 5a). Instead, this translational variant primarily accumulates in the Golgi compartment, as assessed by costaining with the Golgi‐specific protein GM130 (Figure 5b). We do note, however, that a portion of Scimp TV1 is dispersed throughout the cytoplasm (Figure 5b), with this potentially representing a minor endosomal pool. The distinct intracellular localization of Scimp TV1 might contribute to its capacity to enhance CpG DNA responses. To further investigate this, Scimp‐V5 and Scimp TV1‐V5 were expressed in BMMs via retroviral transduction (Supplementary figure 2i, j), treated with an Alexa‐647‐labeled CpG‐containing oligonucleotide DNA, after which Scimp and CpG DNA localization were assessed. Here we found that, similar to our observations in RAW264.7 cells (Figure 5), Scimp TV1 was primarily intracellular, whereas full‐length Scimp accumulated at the cell surface and on filopodia (Supplementary figure 2i). However, there was no obvious colocalization between Scimp TV1 and internalized CpG DNA (Figure 6a). This suggests that the mechanism by which Scimp TV1 promotes CpG DNA responses may be indirect, rather than through enhancing proximal signaling. To further probe this, we examined CpG DNA‐induced signaling by assessing Scimp phosphorylation via SH2 pulldown assays. Scimp‐V5 was phosphorylated at Y58 and Y96 following CpG DNA stimulation, whereas Scimp TV1‐V5 again showed elevated basal phosphorylation and no inducible phosphorylation at either of these sites following treatment with CpG DNA (Figure 6b, c). Similar results for both CpG DNA and Scimp proximity (Supplementary figure 2k), as well as CpG DNA‐inducible Scimp phosphorylation (Supplementary figure 2l), were apparent in Scimp‐deficient RAW264.7 cells reconstituted with Scimp‐V5 and Scimp TV1‐V5. These data support a model in which intracellular Scimp TV1 promotes CpG DNA responses independently of ligand‐induced phosphorylation.

## DISCUSSION

We previously showed that Scimp acts as a scaffold for the LPS‐inducible recruitment of the Src family kinase Lyn to TLR4, thus enabling subsequent TLR4 phosphorylation and inflammatory cytokine production.^25^ Here we identify an alternative translational variant of Scimp, which we refer to as Scimp TV1. We show that Scimp TV1 has a unique subcellular localization, phosphorylation status and role in promoting CpG DNA responses in myeloid cells. Intriguingly, this mechanism of generating a downstream translational variant is conserved in human SCIMP. We note that ectopic expression of both mouse and human SCIMP proteins resulted in the formation of doublet bands in previous publications,^22^, ^24^ with this likely being explained by the alternative translation phenomenon that we report here. However, we have thus far been unable to detect endogenous SCIMP TV1 in human macrophages and we have not investigated functions for the human translational variant. Thus, the biological significance of human SCIMP TV1 remains to be determined. In mouse cells, the initiation of translation at the first start codon in the Scimp mRNA appears to be indispensable for Scimp localization to the plasma membrane (Figure 5a). TMD length, flanking sequences and palmitoylation are all key determinants of pTRAP family member sorting and localization.^36^ A previous study that investigated artificial “TRAP‐like proteins” revealed that proteins with a TMD consisting of 21 hydrophobic amino acids were detectably expressed at the plasma membrane, with removal of two hydrophobic residues resulting in accumulation in the Golgi.^36^ The reduced length of the Scimp TV1 TMD *versus* that of WT Scimp (15 *versus* 21 amino acids; Figure 2a) may thus explain its accumulation in the Golgi and intracellular compartments.

Our observations that Scimp is LPS inducible in CSF‐1‐BMMs (Figure 3c, Supplementary figure S2b) and that Scimp TV1 shows impaired TLR4 binding (Figure 3d, e) suggests that the absence of full‐length Scimp explains the defect in sustained LPS‐inducible IL‐12p40 production that is apparent in macrophages derived from Scimp TV1 mice (Figure 3b). However, Scimp expression alone is unlikely to be sufficient to contribute to sustained LPS signaling, given that GM‐CSF robustly upregulated Scimp expression (Figure 3c),^24^ yet LPS‐inducible cytokine production was not defective in GM‐CSF‐BMMs from Scimp TV1 mice (Figure 4a, b, Supplementary figure 2a). The findings here are congruent with those from a previous study examining Dectin‐1 signaling in *Scimp* ^−/−^ mice.^24^ That study observed no overt phenotype when comparing responses with the Dectin‐1 agonist zymosan in *Scimp* ^−/−^/*MyD88* ^−/−^ bone marrow‐derived dendritic cells *versus* their relevant *MyD88* ^−/−^ controls, whereas there was a reduction in sustained cytokine production in response to this stimulus.^24^ While our study investigated the phenotype of primary cells that were not only deficient in full‐length Scimp but also overexpressing Scimp TV1, it nonetheless seems most likely that the defect in sustained LPS responses reflects a consequence of the former. The contribution of Scimp to sustained cytokine production that was observed in both this study (Figure 3a, b) and a previous one^24^ suggests that inducible production of full‐length Scimp protein may counteract negative feedback loops that dampen inflammatory responses. This may be relevant to endotoxin tolerance, in which prior LPS exposure limits the inflammatory response to a repeat exposure to LPS in both cells and in whole organisms.^37^ Multiple inducible pathways contribute to this tolerance phenotype^37^ and it is possible that upregulation of Scimp expression represents an opposing mechanism that can limit the magnitude and/or duration of endotoxin tolerance.

**Figure 7:**
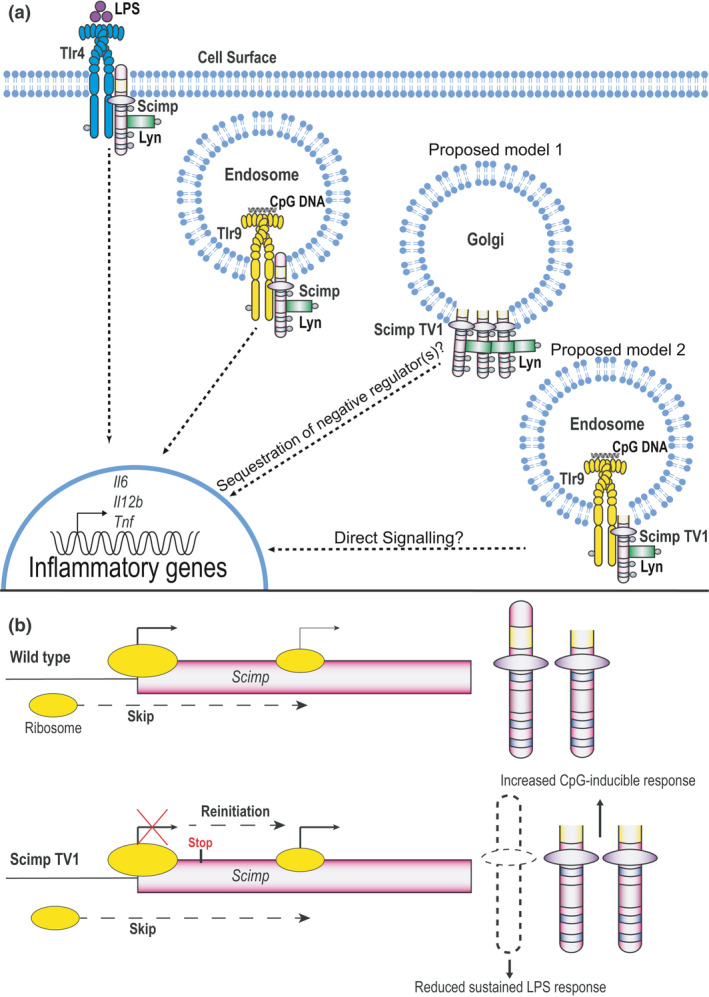
Scimp alternative translation variants and their distinct functions in TLR responses. **(a)** The full‐length Scimp and Scimp TV1 translational variants have unique cellular localizations. Full‐length Scimp promotes phosphorylation‐dependent TLR4 and TLR9 signaling while the intracellular Scimp TV1 variant likely enhances endosomal TLR9 signaling by phosphorylation‐independent mechanisms. The most likely mechanism involved is the sequestration of a negative regulator of TLR9 signaling (Proposed model 1) but it is also possible that Scimp TV1 is involved in direct signaling, for example, at time points not examined in this study (Proposed model 2). **(b)** Scimp can be translated from two separate ATG codons in mice through the process of leaky ribosomal scanning (top). When a stop codon is introduced into the Scimp coding sequence upstream of the second methionine, the expression of full‐length Scimp is ablated and the expression of the downstream Scimp TV1 variant is enhanced, presumably through translation reinitiation (bottom). TLR, Toll‐like receptor.

Compared with GM‐CSF‐BMMs from WT mice, those from Scimp TV1 mice express considerably more Scimp TV1 protein (Figure 3c). This overexpression of Scimp TV1 could enhance Scimp‐dependent responses if indeed Scimp TV1 contributes to TLR signaling, especially considering its heightened basal phosphorylation. Somewhat surprisingly, there was no observable difference between WT and Scimp TV1 GM‐CSF‐BMMs in LPS‐induced cytokine production (Figure 4a, b, Supplementary figure 2a). This would suggest that Scimp is not being recruited or utilized following TLR4 activation in GM‐CSF‐BMMs or that the absence of full‐length Scimp results in redundancy via engagement of another pTRAP family member. Considering the intracellular localization of Scimp TV1, the enhanced CpG DNA response that was apparent in macrophages from Scimp TV1 mice (Figure 4a–i) most likely arises from overexpression of Scimp TV1 rather than deficiency in full‐length Scimp. Consistent with this, we recently reported that knockout of Scimp in RAW264.7 cells impaired CpG DNA responses.^27^ The results found here are also congruent with this previous study, considering that Scimp itself was phosphorylated upon stimulation with CpG DNA (Figure 6b). It is well known that TLR9 recruits many effector molecules in its cellular odyssey and there are numerous mechanisms that facilitate activation of the TLR9 signalosome. An interesting example is UNC93B, which is found in the endoplasmic reticulum in the steady state and shuttles with TLR9 to CpG DNA‐containing compartments, mediating cellular activation.^38^ This protein directly binds the TMDs of the exclusively endosomal TLR3, TLR7 and TLR9 proteins, but does not interact with endosomal TLR4.^38^ Our findings provide additional avenues for exploration of CpG DNA‐specific signaling events through future studies on Scimp TV1. In contrast to full‐length Scimp, we did not observe CpG DNA‐induced phosphorylation of Scimp TV1 (Figure 6b) or obvious colocalization of Scimp TV1 and CpG DNA (Figure 6a). This suggests that Scimp TV1 may promote CpG DNA responses through an indirect mechanism, for example, by sequestration of a negative regulator. Interestingly, Lyn has been shown to negatively regulate CpG DNA responses in dendritic cells by binding to Irf5 and impairing its capacity to drive inflammatory gene expression.^39^ Thus, one possibility is that Scimp TV1 sequesters Lyn to enable maximal CpG DNA responsiveness in GM‐CSF‐BMMs. However, we cannot exclude the possibility that a pool of non‐Golgi‐localized Scimp TV1 (Figures 5b and 6a) does contribute directly to TLR9 signaling, given that our studies only examined acute CpG DNA responses (Figure 6b, c). Figure 7a offers two potential models by which Scimp TV1 may promote TLR9 signaling, with an indirect mechanism seeming the most likely. Whatever the mechanism, it seems likely that there must be some specificity for TLR9 signaling even within the endosomal TLR subfamily, given that responses to the TLR7 agonist imiquimod were not affected in Scimp TV1 GM‐CSF‐BMMs (Figure 4a, b). Considering that Scimp controls responses to many TLRs in murine cells, including TLR3,^27^ more detailed studies on the contribution of Scimp TV1 to signaling from other endosomal TLRs are clearly warranted.

The use of an alternative TSS to generate divergent functions for a single protein has been studied in some biological systems, but not in the context of TLR biology. In dendritic cells, the translation of osteopontin, a pleotropic protein with many proinflammatory functions,^40^ can be initiated downstream of its canonical start site leading to the exclusion of a signal peptide necessary for its secretion.^41^ This intracellular variant of osteopontin was shown to have a distinct role to the full‐length protein, specifically modulating intracellular signaling pathways. In particular, the intracellular form of osteopontin selectively promotes interferon‐α gene expression.^41^ Use of an alternative TSS has not been reported for other pTRAP family members. Rather, diversification of pTRAP protein function has instead been linked to alternative mRNA splicing. Human *LST1* is a pTRAP family member with many splice variants,^42^ of which only the LST1/A isoform has been identified to translate to the protein level at this date. LST1/A itself was not classified as a pTRAP until 2012^43^ and many of its predicted variants lack the TMD and presumably show differential localization.^44^ This suggests that, within the pTRAP family, there is more than one avenue to achieve diversification in isoforms of proteins encoded by a single gene.

When comparing the relative abundance of Scimp and Scimp TV1 in WT cells, full‐length Scimp is clearly the dominantly expressed isoform (Figure 1b). Also of note is the observation that, in the Scimp TV1 mice, more Scimp TV1 is produced than would be expected from leaky scanning alone. There is an introduction of a stop codon upstream of the Scimp TV1 open reading frame (ORF; Figure 1a), which would presumably have meant that ribosomes should disengage, preventing translation of the Scimp TV1 ORF. The fact that there is translation of the Scimp TV1 ORF at such levels indicates that there is a reinitiation of translation occurring after it has passed the inserted stop codon. This may reflect the ribosome staying bound to the mRNA and that translation initiation factors remain bound or are re‐recruited to the ribosome. This is known to occur following translation of small ORFs,^45^, ^46^ such as the one generated upstream of the Scimp TV1 ORF. The expression of the two Scimp variants following the Kozak “correction” (Figure 2e) indicates that this expression pattern seen in WT cells predominantly occurs via leaky scanning by the ribosome, whereas the relative increased expression of Scimp TV1 in Scimp TV1 BMMs is likely because of a translation reinitiation event (Figure 7b). Of note, the Kozak correction did not result in the complete ablation of the alternative translational variant arising from leaky scanning (Figure 2e), which is consistent with findings from others.^47^

What remains unknown is whether there is any form of regulatory control that modulates this ribosomal slippage in Scimp‐expressing cells and whether there is any circumstance where there is a shift in expression between the two translational variants. Interestingly, when comparing the abundance of Scimp *versus* Scimp TV1 in CSF‐1‐ and GM‐CSF‐BMMs, the expression of the full‐length variant appears to be more pronounced in the GM‐CSF‐BMMs (Figure 3c). While this suggests a preference for expression of full‐length Scimp in an inflammatory context, the mechanism that promotes this is unclear. It will be of interest in the future to determine whether there are specific conditions favoring translation from the second ATG to generate enhanced Scimp TV1 expression. In summary, in this report we have described a novel translational variant of the pTRAP family member Scimp, showing that it has a unique cellular localization and function when compared with its full‐length counterpart. This alternative usage of a downstream translational start site suggests a novel mechanism whereby a single adaptor protein can contribute to fine‐tuning responses to multiple receptors that operate through disparate signaling mechanisms (Figure 7a).

## METHODS

### Animal ethics and handling

All animal studies were approved by The University of Queensland Animal Ethics Committee (AEC Approval numbers: IMB/118/15 and IMB/123/18). Scimp‐TV1 mice on a C57BL/6 background were generated by the Monash gene targeting facility, through pronuclear injection of a Scimp sgRNA (GT TGG TGG AGG GAC AAC TTC TGG), a repair oligo that inserts a stop codon after the seventh amino acid (TCATGCCTCAGTCTTTGTTCTTCCTAGGCTCCCACAGCAATGAGTTGGTGGAGGGACAAC‐TGAATTCTTCTGGATCATCTTAGCTATGTCCATCATCTTCATCTCCCTGGTCCTGGGTCTCATCCTG) and a CRISPR‐associated protein 9‐encoding RNA. The sequence variant is thus technically called NC_000077.7:g.70691607_70691608insAATTCAG (NM_001045526.2:c.118_119insCTGAATT). After germline transmission was confirmed, two independent founders were further crossed on to the C57BL/6 background prior to assessing Scimp expression and functions.

### Cell culture and reagents

*Scimp*‐deficient RAW264.7 cells^25^ were reconstituted with V5‐tagged Scimp constructs or empty vector controls, and cultured in Roswell Park Memorial Institute (RPMI)‐1640 medium (Gibco, Billings, MT, USA) supplemented with 2 mM GlutaMAX (Life Technologies, Carlsbad, CA, USA), 5% fetal bovine serum (FBS, Gibco), 50 U mL^−1^ penicillin (Life Technologies) and 50 µg mL^−1^ streptomycin (Life Technologies) (RAW complete media). For selection of RAW264.7 cell lines stably overexpressing specific proteins, 2 μg mL^−1^ blasticidin (Merck, Kenilworth, NJ, USA) was added to RAW complete media. HEK293T cells (ATCC, Manassas, VA) were cultured in Dulbecco’s modified Eagle medium (Gibco) containing 2 mM GlutaMAX (Life Technologies) supplemented with 10% FBS, 50 U mL^−1^ penicillin and 50 μg mL^−1^ streptomycin (HEK complete media). Human monocytic THP‐1 cells (ATCC) were cultured in RPMI medium supplemented with 2 mM GlutaMAX (Life Technologies), 10% FBS, 50 U mL^−1^ penicillin, 50 µg mL^−1^ streptomycin, 1 mM sodium pyruvate (Gibco) and 10 mM 4‐(2‐hydroxyethyl)‐1‐piperazineethanesulfonic acid (HEPES; Gibco; THP‐1 complete media). THP‐1 cells were differentiated using 30 ng mL^−1^ phorbol‐12‐myristate‐13‐acetate (Sigma‐Aldrich, St. Louis, MO, USA) for 48 h prior to experiments. Platinum‐E retroviral packaging (PlatE) cells^48^ were cultured in the presence of Dulbecco’s modified Eagle medium (Gibco) containing 2 mM GlutaMAX (Life Technologies) supplemented with 10% FBS, 50 U mL^−1^ penicillin and 50 μg mL^−1^ streptomycin (PlatE complete media). Interferon‐γ (Bio‐scientific, Kirrawee, Australia) was used to stimulate cells at a concentration of 5 ng mL^−1^. GM‐CSF (Miltenyi Biotec, Bergisch Gladbach, Germany) was dissolved in water to a concentration of 25 μg mL^−1^ and stored at −20°C before being thawed and diluted in relevant media and used at a concentration of 10 ng mL^−1^. Doxycycline (Sigma‐Aldrich) was dissolved in water to a concentration of 10 mg mL^−1^ and stored at −20°C before being thawed and diluted in relevant media to be used at concentrations of 100 ng mL^−1^.

### Primary macrophage culture

Murine BMMs were generated by growth factor‐mediated differentiation of hematopoietic stem cells isolated from femur and tibia bone marrow extracted from 8–12‐week‐old male and female C57BL/6 mice. Post‐euthanasia, tibias and femurs were collected and sterilized using 70% ethanol prior to flushing of the bone marrow with a 27‐gauge needle (Terumo, Tokyo, Japan). Once extracted, bone marrow from all four bones was cultured in eight square 10‐cm^2^ bacteriological plastic culture dishes (formerly Sterilin, Thermo Fisher Scientific, Waltham, MA, USA) for 6–7 days in the presence of recombinant human colony‐stimulating factor 1 (CSF‐1), used at either 1 × 10^4^ U mL^−1^ (Chiron, Emeryville, CA, USA) or 150 ng mL^−1^ (The University of Queensland Protein Expression Facility, Australia). CSF‐1‐BMMs were cultured in the presence of RPMI‐1640 medium (Gibco) supplemented with 2 mM GlutaMAX (Life Technologies), 10% FBS, 50 U mL^−1^ penicillin, 50 µg mL^−1^ streptomycin and CSF‐1 (BMM Complete media). GM‐CSF‐BMMs were cultured in the presence of RPMI‐1640 supplemented with 2 mM GlutaMAX (Life Technologies), 10% FBS, 50 U mL^−1^ penicillin, 50 µg mL^−^1 streptomycin and 10 ng mL^−1^ GM‐CSF (Miltenyi Biotec, Bergisch Gladbach, Germany). CSF‐1‐BMMs and GM‐CSF‐BMMs were harvested on day 6 and plated in BMM complete media for experimentation on day 7, unless described otherwise.

### Mammalian expression and lentiviral expression constructs

The mouse Scimp‐V5 construct has previously been described.^25^ Scimp TV1‐V5 was generated by PCR from the Scimp‐V5 construct using a forward primer that excludes the first 39 base pairs (first 13 amino acids) and topoisomerase‐mediated cloning into pEF6/V5‐His TOPO TA. The Scimp‐M14A‐V5 and Scimp‐M14V‐V5 constructs were generated from Scimp‐V5 constructs using PCR mutagenesis followed by restriction enzyme digest and ligation into a pEF6/V5‐HIS TOPO TA. Scimp_pMIGRMCS_GFP constructs were generated by restriction enzyme digest and ligation from Scimp_pEF6 constructs into the empty vector pMIGRMCS_GFP.^49^ hIL‐23p19 was PCR cloned into pEF6 from human monocyte‐derived macrophages complementary DNA (cDNA). mGas7 was PCR cloned into pEF6 from mouse brain cDNA, incorporating only the N terminus of the protein (amino acids 1–159). All constructs were cloned without their native stop codons to incorporate a C‐terminal V5 tag and all were confirmed by DNA sequencing. The lentiviral system utilizing pF_TRE3G_PGK_puro (hereafter pLenti_EV) was kindly provided by James Murphy, The Walter and Eliza Hall Institute of Medical Research, Melbourne, Australia, and used for doxycycline‐inducible gene expression. Human SCIMP‐Myc constructs^22^ were used as a template to generate SCIMP‐M1V‐Myc and SCIMP‐M12V‐Myc constructs by PCR mutagenesis, after which they were subcloned into pLenti_EV. All constructs are listed in Supplementary table 1 and cloning primers used are listed in Supplementary table 2.

### Toll‐like receptor agonists

The TLR4 agonist LPS was chromatographically purified from *Salmonella enterica* serotype Minnesota Re 595 (Cat: L2137, Sigma‐Aldrich) and was used at concentrations listed in individual figures. The TLR7 agonist imiquimod (R837; Invitrogen, Waltham, MA, USA) was diluted in sterile water and used at a final concentration of 20 μg mL^−1^. Pam3[Cys‐Ser‐(Lys)4] hydrochloride (Pam3CSK4; Merck) was used as a TLR1/2 agonist and diluted in sterile water to be used at a final concentration of 15 ng mL^−1^. Mouse‐stimulatory CpG‐containing DNA (CpG 1688) was synthesized with a phosphorothioate backbone (Merck) using the following sequence: 5′‐TCCATGACGTTCCTGATGCT‐3′. CpG DNA was reconstituted in sterile water before being diluted further in relevant media and used at concentrations listed in individual figures. An Alexa‐647‐conjugated CpG 1668 oligonucleotide was synthesized to contain a phosphorothioate backbone (IDT, Coralville, IA, USA).

### Transient transfection

HEK293T cells (0.6 × 10^6^) were plated in 6‐well plates in HEK complete media, then 24 h later transfected with pEF6 expression constructs using Lipofectamine 2000 (Invitrogen). 2 μg of DNA constructs were mixed with 350 μL of Opti‐MEM (Gibco); 7 μL of Lipofectamine 2000 was mixed into 350 μL of Opti‐MEM and left at room temperature for 4 min. Opti‐MEM‐containing DNA and Opti‐MEM‐containing Lipofectamine were mixed and incubated at room temperature for 20 min. The DNA and Lipofectamine Opti‐MEM mix were added to HEK293T cells dropwise and returned to the 37°C incubator. At 24 h after transfection, cells were lysed and cell extracts were immunoblotted as detailed below.

### RNA purification and cDNA synthesis

Cells were lysed in 350 μL TRIzol (Invitrogen) or RLT buffer (Qiagen, Hilden, Germany), after which total RNA was extracted using the relevant RNA extraction kit (Zymo, Irvine, CA for TRIzol; Qiagen for RLT), as per the manufacturer’s guidelines and quantified using the ND1000 nano‐drop spectrophotometer (Thermo Fisher Scientific). Contaminating genomic DNA was removed using on‐column DNase digestion (Qiagen) during RNA extraction. 1000 ng of RNA was incubated at 65°C for 5 min in a cocktail containing 500 ng oligo dT primers (Merck) and 10 mM dNTP followed by 1‐min incubation on ice. The RNA/oligo dT dimer was then reverse transcribed using a cocktail containing Superscript III, first‐strand reaction buffer and 0.1 M DTT (Invitrogen) while being incubated at 50°C for 50 min, then 70°C for 10 min. A no RT control was generated using RNA collected from all samples in a set that was then treated as above but without the incorporation of Superscript III into the reverse transcription cocktail. cDNA samples were diluted in ultrapure DNase/RNase free water (Gibco) and stored at −20°C.

### Gene expression analysis via RT‐qPCR

RT‐qPCR was performed in 384‐well plates (Applied Biosystems, Waltham, MA, USA) with each well containing 5 μL SYBR Green PCR Master Mix (Applied Biosystems), 2 μL of forward and reverse primers at 2 μM (Supplementary table 3), 1 μL of DNase/RNase‐free water (Gibco) and 2 μL of diluted cDNA. All samples were run in triplicate wells for each gene of interest and levels of mRNA were quantified in a Viia7 RT‐PCR system (Applied Biosystems). Gene expression was normalized to the expression of the housekeeping gene hypoxanthine phosphoribosyltransferase (*Hprt*) and analyzed using the delta Ct Method.

### Gene overexpression in THP‐1 by lentiviral transduction

Lentiviral transduction was used for gene overexpression in THP‐1 cells. HEK293T cells were plated overnight at 2 × 10^6^ on a 10‐cm tissue culture plate in 10 mL media, using one plate per transfection. A no‐transfection control was also included. A solution of 60 µL Lipofectamine 2000 (Invitrogen) in 1.5 mL Opti‐MEM (Gibco) was made up for each transfection and incubated at room temperature for 5 min. This solution was then added to 1.5 mL of Opti‐MEM (Gibco) containing 1 μg of each of the lentiviral packaging and envelope plasmids pCMV‐dR8.2 dvpr and pCMV_VSV‐G (Addgene, Watertown, MA, USA), along with the appropriate transfer plasmid for gene overexpression (see Supplementary table 1). This mixture (~3 mL) was incubated at room temperature for 20 min, then added to cultured HEK293T cells and left to incubate at 37°C for 8 h. The media was then removed and replaced with 6 mL complete RPMI media overnight. In the morning, 2 × 10^6^ (nondifferentiated) THP‐1 cells were plated in non‐TC 6‐well plates (Thermo Fisher Scientific). Viral supernatants (~6 mL) were collected from transfected HEK293T cells, then filtered through a 0.45‐μm Millex‐HV polyvinylidene fluoride syringe filter (Merck) into 15‐mL Falcon tubes (BD Biosciences, Franklin Lakes, NJ), with 60 ng (4 µL) polybrene (Merck) added to each supernatant. 6 mL of THP‐1 complete media was then added onto the transfected HEK293T cells, after which cells were incubated for a further 24 h to be used for the second infection. Viral supernatants were added to THP‐1 cells and plates were centrifuged at 1000*g* at 35°C for 2 h. Infected THP‐1 cells were returned to the incubator and incubated for a further 24 h. Following this, the process was repeated, with the removal, filtering, addition and spinfection of viral supernatants on to THP‐1 cells. Following the second incubation for 24 h, THP‐1 cells were harvested and washed with THP‐1 complete media twice before being replated in a T75 flask and left to recover for 24 h. THP‐1 cells were then placed under 1 µg mL^−1^ puromycin (Sigma‐Aldrich) selection for 48 h. Subsequently, for validation and future experiments, THP‐1 cells were plated and differentiated with phorbol‐12‐myristate‐13‐acetate, as described above. Inducible overexpression of proteins (Scimp‐Myc, Scimp M1V‐Myc and Scimp M12V‐Myc) was assessed following addition of 100 ng mL^−1^ doxycycline (Sigma‐Aldrich) for 24 h.

### MTT assays

For experiments where ELISAs were performed on different cell populations, plating density was assessed by concurrently plating 4 × 10^4^ cells in 96‐well plates. Cells were left to adhere overnight, then incubated with 1 mg mL^−1^ MTT reagent (Sigma‐Aldrich), diluted in the appropriate complete media. Cells were left at 37°C for 1–3 h. The MTT media was removed and formazan crystals were dissolved in 100% isopropanol. Once the formazan precipitate was fully dissolved, the absorbance at 510 nm was read using a PowerWave XS (BioTek, Winooski, VT, USA) or Infinite M Plex (Tecan, Mannedorf, Switzerland) plate reader.

### Stable gene overexpression by electroporation in RAW264.7 cells

Scimp‐reconstituted RAW264.7 cells were generated as previously described.^27^ In brief, RAW264.7 cells were resuspended in 0.4‐cm electroporation cuvettes at 4 × 10^6^ cells/390 µL, with 10 µg of plasmid DNA at a final volume of 400 µL. Cells were electroporated at 240 V, 1000 µF, and ∞ Ω. After electroporation, cells were washed twice with RAW complete media and then plated. Cells were left to recover for 24 h prior to the addition of blasticidin (2 µg mL^−1^). Cells were cultured in the presence of blasticidin (2 µg mL^−1^) for ~2 weeks to ensure stable integration of plasmid DNA.

### Gene overexpression by retroviral transduction in bone marrow‐derived macrophages

2 × 10^6^ PlatE cells were plated in 10‐cm dishes and left to adhere overnight. PlatE cells were transiently transfected with empty vector pMIGRMCS_GFP or various Scimp_pMIGRMCS_GFP constructs (Supplementary table 1) using Lipofectamine 2000 (Invitrogen). 24 h after transfection, cells were washed and incubated at 32°C for 48 h to facilitate virus production. At the same time, mouse bone marrow was collected from C57BL/6 mice, plated in BMM complete media and incubated at 37°C for 48 h. 10 mL of viral supernatant was collected from transfected PlatE cells, filtered for retrovirus using 0.45‐µm Millex‐HV polyvinylidene fluoride syringe filters (Merck) into labeled 15‐mL Falcon tubes. 20 mM HEPES (Gibco), 60 ng polybrene (4 mg mL^−1^ stock, Merck) and 10^4^ U CSF‐1 were added to each supernatant. BMM progenitors were collected from plates and added equally to retroviral supernatants before being aliquoted onto non‐tissue culture 6‐well plates. Plates were centrifuged at 1000*g* at 35°C for 2 h to facilitate viral uptake. At 48 h post‐infection, the media was replaced with BMM complete media. BMMs were collected on day 6 and assessed for transduction efficiency by measuring GFP expression using flow cytometry, before being plated for further experiments.

### Whole‐cell extracts and immunoblotting

Whole lysates were collected by lysing cells in radioimmunoprecipitation assay buffer (50 mM Tris pH 7.4, 150 mM NaCl, 1 mM ethylenediaminetetraacetic acid, 1% Triton X‐100, 1% sodium deoxycholate, 0.1% sodium dodecyl sulfate) supplemented with cOmplete, ethylenediaminetetraacetic acid‐free Protease Inhibitor Cocktail (Sigma‐Aldrich) and PhosSTOP phosphatase inhibitor (Sigma‐Aldrich). Immunoblotting was performed by electrophoresing equal amounts of protein (determined by Bicinchoninic acid assays) through precast BOLT gels (Invitrogen), followed by turbo transfer at 25 V for 9 min onto nitrocellulose membranes (Bio‐Rad, Hercules, CA, USA). Membranes were then blocked in 5% skim milk in Tris‐buffered saline containing 0.05% Tween 20, followed by probing with the indicated antibodies (Supplementary table 4). Proteins were visualized using Clarity ECL (Bio‐Rad) and ChemiDoc. Membranes were either stripped using ReBlot Plus Strong Solution (Merck) at room temperature for 15 min or quenched with hydrogen peroxide 30% (Merck) at 37°C for 20 min, enabling reprobing of blots.

### Immunoprecipitation assays

Cells were plated in complete media, left to adhere overnight and then stimulated for designated periods with specific concentrations of LPS, as indicated in individual figure legends. Media was removed and cells were harvested in chilled phosphate‐buffered saline (PBS) with a cell scraper. Cells were pelleted and lysed in 700 µL co‐immunoprecipitation lysis buffer [20 mM TRIS (pH 7.4), 150 nM NaCl, 1% NP40 and 5% glycerol] supplemented with cOmplete, ethylenediaminetetraacetic acid‐free Protease Inhibitor Cocktail (Sigma‐Aldrich) and PhosSTOP phosphatase inhibitor (Sigma‐Aldrich). Lysates were passed through a 28‐gauge needle and centrifuged at 10 000*g* for 15 min. 400 µL of lysate was added to columns containing 20 µL of Pierce protein G plus agarose beads (Life Technologies) and 2 µL of capture antibody (Supplementary table 4). Columns were incubated at 4°C while rotating for 1 h. Columns were centrifuged to remove the after‐bind mix, then washed three times with co‐immunoprecipitation lysis buffer. Bound samples were boiled using Bolt LDS Sample Buffer (Life Technologies) and NuPAGE Sample Reducing Agent (Life Technologies) before being eluted from columns and immunoblotted.

### SH2 domain pulldown assays

Cells were plated in complete media, left to adhere overnight, then stimulated as indicated in figure legends. Media was removed and cells were harvested in chilled PBS with a cell scraper. Cells were pelleted and lysed in 200 µL co‐immunoprecipitation lysis buffer supplemented with cOmplete, ethylenediaminetetraacetic acid‐free Protease Inhibitor Cocktail (Sigma‐Aldrich) and PhosSTOP phosphatase inhibitor (Sigma‐Aldrich). Lysates were passed through a 28‐gauge needle and centrifuged at 10 000*g* for 15 min. 30 µL of sample was added to columns that contained 200 µg (20 µL of 10 µg per µL stock solution) of SH2 probe [SH2 domain of either human CSK, GRB2 or SLP65 conjugated to GST and bound to Pierce protein G plus agarose beads (Life Technologies) as described in Luo *et al.* ^26^]. SH2 probes were stored at −20°C prior to use. Columns were incubated overnight at 4°C while rotating, then centrifuged to remove after‐bind mix and washed three times with co‐immunoprecipitation lysis buffer. Bound samples were boiled using Bolt LDS Sample Buffer (Life Technologies) and NuPAGE Sample Reducing Agent (Life Technologies) before being eluted from columns and immunoblotted.

### ELISA

Levels of secreted mouse IL‐6, IL‐12p40 and Tnf were assessed via sandwich ELISA using antibodies listed in Supplementary table 4. The 96‐well ELISA plates (Nunc, Rochester, NY, USA) were coated with capture antibody (diluted in 0.1 M sodium bicarbonate, pH 8.35) overnight. Plates were washed twice (with PBS containing 0.05% Tween), before being blocked with 10% FBS in PBS for 2 h at 37°C or overnight at 4°C. Plates were washed before samples and standards (diluted in the relevant complete media) were added and incubated for 2 h at 37°C or overnight for 4°C. Plates were then sequentially incubated and washed with secondary antibody (diluted in 10% FBS in PBS) for 1 h at 37°C, followed by extra‐avidin (1:1000 dilution in 10% FBS in PBS) for 20 min at 37°C. After further washing, the TMB substrate (BD OptEIA; BD Biosciences) was added. Reactions were stopped using 2 M sulfuric acid and absorbance at 450 nm was read using a plate reader (Infinite M Plex, Tecan). Cytokine levels were calculated by extrapolation from a sigmoidal curve analysis of the standards.

### Confocal microscopy

2 × 10^5^ cells were plated on coverslips in 24‐well plates in the appropriate complete media and left overnight. Following stimulation as described in the figure captions, cells were washed twice with PBS before being fixed in 4% PFA (Sigma‐Aldrich) for 30 min. Once fixed, cells were washed of PFA with PBS twice before permeabilization using 0.1% Triton X (Sigma‐Aldrich) in PBS for 5 min. Cells were washed of Triton X with PBS three times before being blocked in 2% bovine serum albumin (Sigma‐Aldrich) in PBS for 30 min, then washed and stained with primary antibody (Supplementary table 4) diluted in 2% bovine serum albumin in PBS. Cells were again washed and then stained with 4′,6‐diamidino‐2‐phenylindole (20 ng mL^−1^ diluted in 2% bovine serum albumin in PBS), wheat germ agglutinin‐Alexa Fluor 647 (1.25 µg mL^−1^ diluted in 2% bovine serum albumin in PBS), rat anti‐mouse Alexa Fluor 568 (1:5000) or rat anti‐mouse Alexa Fluor 647 (1:400; Supplementary table 4). The coverslips were mounted using IMBiol mounting media (IMB) and slides were viewed using a Zeiss Axiovert 200 Upright Microscope Stand with LSM 710 Meta Confocal Scanner and spectral detection with 63× magnification (Zeiss, Oberkochen, Germany). Images were processed with Fiji (ImageJ).

### Statistical analyses

Quantitative data acquired from each independent experiment were averaged across technical replicates, after which data from independent experiments were combined and represented as mean ± standard error of the mean (s.e.m.) of *n* (*n* = number of independent experiments). Data with *n* < 3 were represented as mean ± range of the data. Statistical analyses on data combined from ≥ 3 independent experiments were performed using GraphPad Prism software. Significance was determined using *t*‐tests, one‐way or two‐way analysis of variance (ANOVA) and Bonferroni *post hoc* multiple comparisons testing, as described in the individual figure captions.

## AUTHOR CONTRIBUTIONS

**James EB Curson:** Conceptualization; Data curation; Investigation; Methodology; Project administration; Visualization; Writing – original draft; Writing – review & editing. **Lin Luo:** Conceptualization; Data curation; Funding acquisition; Investigation; Methodology; Supervision; Writing – review & editing. **Liping Liu:** Methodology; Resources. **Belinda J Burgess:** Conceptualization; Investigation; Methodology; Visualization. **Nilesh J Bokil:** Conceptualization; Supervision. **Adam A Wall:** Conceptualization; Methodology. **Tomas Brdicka:** Resources. **Ronan Kapetanovic:** Conceptualization; Methodology; Supervision; Writing – review & editing. **Jennifer L Stow:** Conceptualization; Funding acquisition; Supervision; Writing – review & editing. **Matthew J Sweet:** Conceptualization; Funding acquisition; Project administration; Supervision; Writing – original draft; Writing – review & editing.

## CONFLICT OF INTEREST

The authors declare that they have no conflicts of interest with the contents of this article.

## Supporting information

Supplementary Material

## Data Availability

The data that support the findings of this study are available from the corresponding author upon reasonable request.
