# Supplementary material for: An alternative downstream translation start site in the non‐TIR adaptor Scimp enables selective amplification of CpG DNA responses in mouse macrophages
Source: Immunol Cell Biol. 2022 Mar 22;100(4):267–84. doi: 10.1111/imcb.12540 (PMC9544816; doi:10.1111/imcb.12540)
Supplement: Supplementary file 1 — Supplementary Material [file IMCB-100-267-s001.docx]

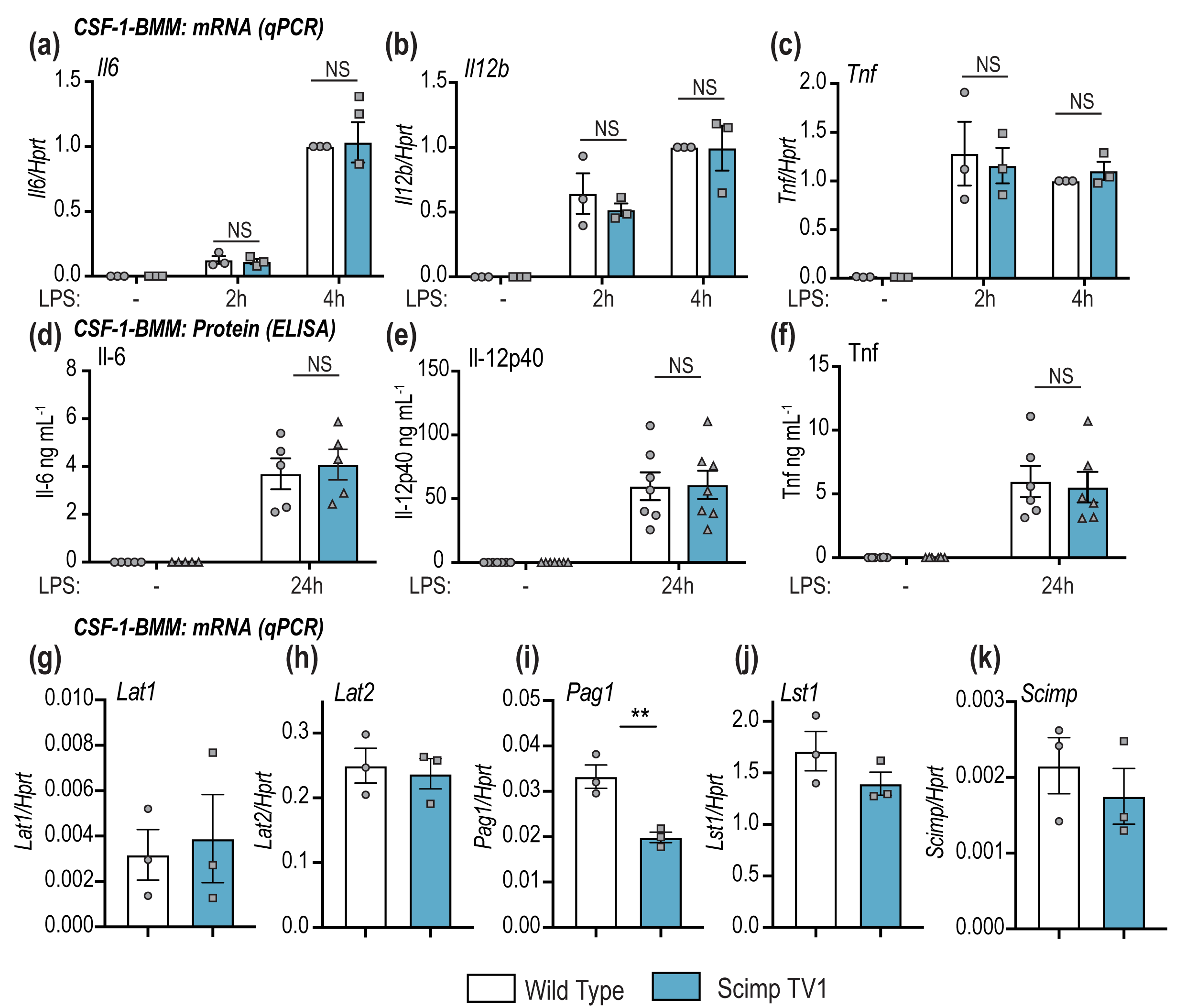


**Supplementary figure 1. Scimp TV1 CSF-1- BMM exhibit no defect in LPS-inducible Il-12p40, Il-6 or Tnf production.**

**(a-c)** Wild type and Scimp TV1 CSF-1-BMM were treated with LPS (10 ng mL^-1^) for 2 or 4 h. Total RNA was collected and assessed for *Il6*, *IL12b* and *Tnf* mRNA expression via RT-qPCR. Data (mean ± SEM, n=3 mice per genotype) are combined from three independent experiments and are normalized to the wild type 4 h LPS time point. **(d-f)** Wild type and Scimp TV1 CSF-1-BMM were treated with LPS (10 ng mL^-1^) for 24 h. Supernatants were collected and assessed for Il-6, Il-12p40 and Tnf production via ELISA. Data (mean ± SEM, n=5-7 mice per genotype) are combined from 5 to 7 independent experiments. **(g-k)** Wild type and Scimp TV1 CSF-1-BMM were plated and left unstimulated. Total RNA was collected and *Lat1* **(g)**, *Lat2* **(h)**, *Pag1* **(i)**, *Lst1* **(j)** and *Scimp* **(k)** mRNA levels, relative to *Hprt*, were determined by qPCR. Data (mean ± SEM, n=3 mice per genotype) are combined from three independent experiments. **(a-f)** Statistical analyses were performed using two-way ANOVA, followed by Bonferroni’s multiple comparison test using GraphPad Prism (NS, non-significant). **(g-k)** Statistical significance was determined using an unpaired *t*-test using Graphpad Prism (***P* < 0.01).

**
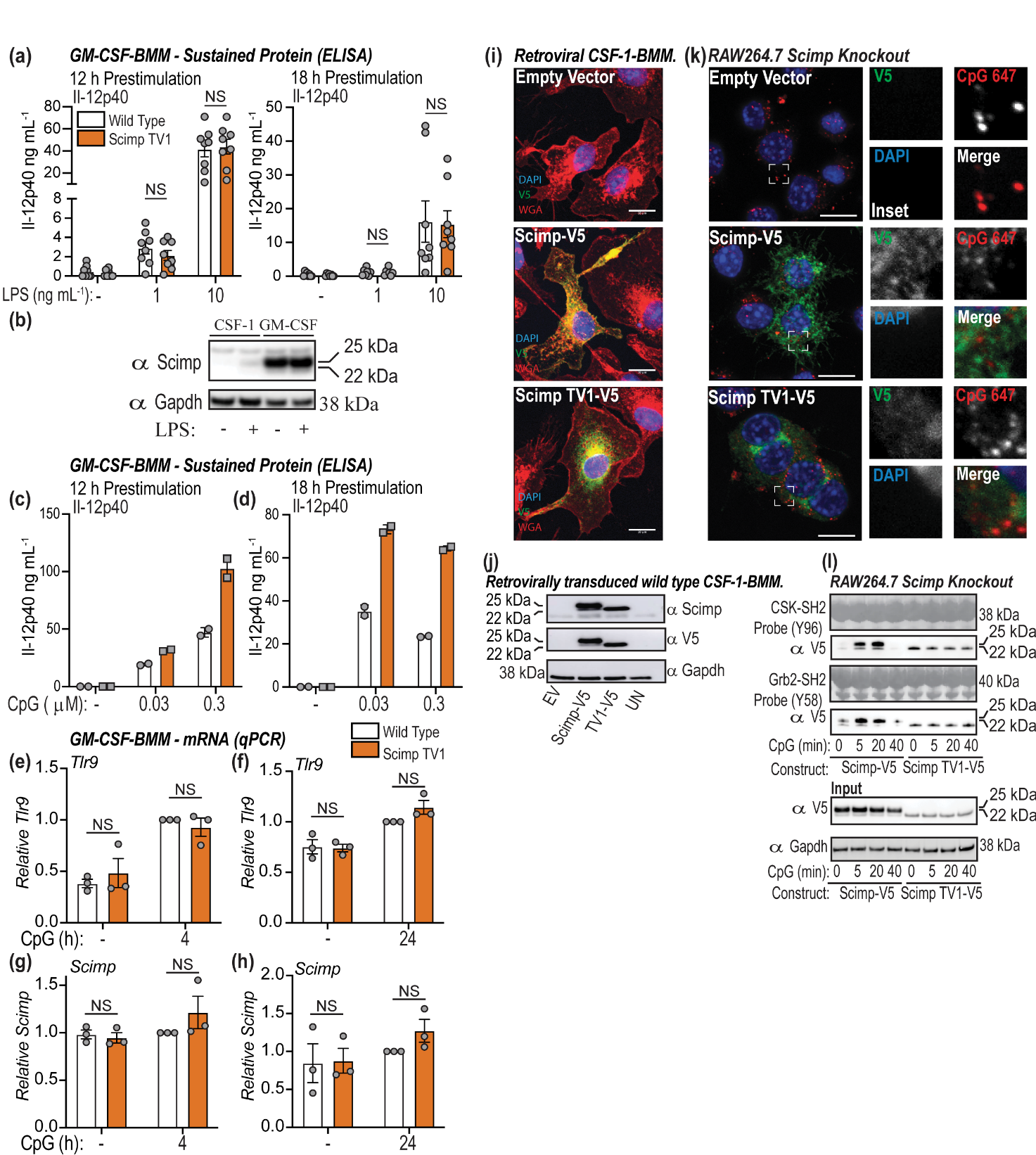
**

**Supplementary figure 2. Scimp overexpression in wild type BMM.**

**(a)** GM-CSF-BMM from wild type and Scimp TV1 mice were stimulated with LPS (1 ng mL^-1^ or 10 ng mL^-1^) for the indicated time points, washed, then replated in fresh media for a further 24 h. Supernatants were collected and assessed for Il-12p40 production by ELISA. Data (mean ± SEM, n= 7-8 mice per genotype) are combined from 7-8 independent experiments. **(b)** Wild type BMM were differentiated with either CSF-1 or GM-CSF for 6 d before stimulation with LPS (100 ng mL^-1^) for 24 h. Cells were lysed and whole cell lysates were assessed for Scimp expression via western blot. Similar results were observed in 3 independent experiments using 3 wild type C57Bl/6 mice. **(c, d)** GM-CSF-BMM from wild type and Scimp TV1 mice were stimulated with CpG (0.03 μM or 0.3 μM) for the indicated time points, washed, then replated in fresh media for a further 24 h. Supernatants were collected and assessed for Il-12p40 production by ELISA. Data show mean ± range (n=2 technical replicates of one experiment), with similar findings observed in four independent experiments collected from 4 mice per genotype (shown in **Figure 4i-h**). **(e-h)** GM-CSF-BMM from wild type and Scimp TV1 mice were stimulated with CpG DNA (0.3 μM) for 4 h **(e, g)** or 24 h **(f, h)**. Total RNA was collected and assessed for *Tlr9* and *Scimp* mRNA levels by RT-qPCR. Data (mean ± SEM, n=3 mice per genotype) are combined from 3 independent experiments. **(i-j)** Wild type CSF-1-BMM were retrovirally transduced with either an empty vector (EV), Scimp-V5 or Scimp TV1-V5 expression constructs and assessed for V5-tagged Scimp via immunofluorescence microscopy (anti-V5/Scimp: green; WGA: red; DAPI: blue; scale bars: 10 μm) **(i)** and western blot **(j)**. Similar results were observed in 2 independent experiments in which a total of 2 wild type C57Bl/6 mice were retrovirally transduced (1 mouse per experiment). **(k, l)** Scimp knock-out RAW264.7 cells were reconstituted with an empty vector, Scimp-V5 or Scimp TV1-V5. **(k)** Cells were treated with 0.3 μM fluorescently-labelled CpG DNA (CpG 647). Cellular localisation of V5-tagged Scimp proteins and CpG DNA were assessed via immunofluorescence microscopy (anti-V5/Scimp: green; CpG 647: red; DAPI: blue; scale bars: 10 μm). Similar results were observed in 3 independent experiments. **(l)** SH2 pulldown assays were used to assess CpG DNA-inducible phosphorylation of Y58 (Grb2-SH2) and Y96 (CSK-SH2). Data are from a single experiment. Statistical significance in **(a, e-h)** was determined using two-way ANOVA, followed by Bonferroni’s multiple comparison test using GraphPad Prism (NS, non-significant).

**Supplementary table 1. Constructs**

| **Construct Name** | **Expression backbone** | **Tag** |
| --- | --- | --- |
| mScimp_V5_pEF6 | pEF6-V5/His | V5 |
| mScimp_TV1_V5_pEF6 | pEF6-V5/His | V5 |
| mScimp_M14A_V5_pEF6 | pEF6-V5/His | V5 |
| mScimp_M14V_V5_pEF6 | pEF6-V5/His | V5 |
| hIL-23p19_V5_pEF6 | pEF6-V5/His | V5 |
| mGas7_V5_pEF6 | pEF6-V5/His | V5 |
| mScimp_Kozak_S2G_V5_pEF6 | pEF6-V5/His | V5 |
| mScimp_V5_pMIGRMCS_GFP | pMIGRMCS_GFP | V5 |
| mScimp_TV1_V5_pMIGRMCS_GFP | pMIGRMCS_GFP | V5 |
| SCIMP_Myc_Lenti_MCS | pF_TRE3G_PGK_puro | Myc |
| SCIMP _M1V_Myc_Lenti_MCS | pF_TRE3G_PGK_puro | Myc |
| SCIMP _M12V_Myc_Lenti_MCS | pF_TRE3G_PGK_puro | Myc |

**Supplementary table 2. Cloning primers**

| **Gene** | **FWD Primer (5’-3’)** | **REV Primer (5’-3’)** | **Purpose** |
| --- | --- | --- | --- |
| *Scimp V5 total* | CCTTGCGCTTAAGGAGC | GCCCTCTAGACTCGAGCG | Amplification of Scimp from pEF6 construct during PCR mutagenesis of M14A/V constructs |
| *Scimp TV1* | AGCTATGTCCATCATCTTCATCTCC | AAACGAGCTGTGTAAACCCAC | Removal of first 13 amino acids via amplifying Scimp from the second methionine onwards |
| *Scimp M14V* | ATCATCTTAGCTGTGTCCATCATC | GATGATGGACACAGCTAAGATGAT | Generation of a point mutant incapable of generating Scimp TV1 |
| *Scimp M14A* | TGGATCATCTTAGCTGCGTCCATCATCTTC | GAAGATGATGGACGCAGCTAAGATGATCCA | Generation of a point mutant incapable of generating Scimp TV1 |
| *Lenti insert total* | GAAGGTGGAGAGAGAGACAGAG | CTAGGGGAGGAGTAGAAGGT | Lenti_MCS sequencing primers used to amplify SCIMP from Lenti construct during PCR mutagenesis |
| *SCIMP M1V* | CCGCTCACATGTGGATACTTTCAC | GTGAAAGTATCCACATGTGAGCGG | Generation of a point mutant incapable of generating full-length SCIMP translational variant |
| *SCIMP M12V* | ATTCCACTGCAGTGAGCTGGTGGAGG | CCTCCACCAGCTCACTGCAGTGGAAT | Generation of a point mutant incapable of generating SCIMP TV1 |
| *Scimp Kozak S2G* | GCCACCATGGGTTGGTGGAGGGA | AAACGAGCTGTGTAAACCCAC | Generation of a mutant with an optimised Kozak sequence. To match the optimal sequence, a missense mutation of the 4^th^ bp was required, resulting in S2G mutation. |

**Supplementary table 3. RT-qPCR primers for mouse genes**

| **Gene** | **Forward Primer (5’-3’)** | **Reverse Primer (5’-3’)** | **Amplicon size (bp)** |
| --- | --- | --- | --- |
| *Il6* | CTGCAAGAGACTTCCATCCAGTT | GAAGTAGGGAAGGCCGTGG | 70 |
| *Il12b* | GGAAGCACGGCAGCAGAATA | AACTTGAGGGAGAAGTAGGAATGG | 180 |
| *Tnf* | CATCTTCTCAAAATTCGAGTGACAA | TGGGAGTAGACAAGGTACAACCC | 175 |
| *Hprt* | GCAGTACAGCCCCAAAATGG | AACAAAGTCTGGCCTGTATCCAA | 85 |
| *Scimp* | ACCTAGCCCCACAGGAAGC | GTGGAGCCCGAGATAGCAAA | 97 |
| *Lat1* | GAGCTGGCCTCTGTGAACTC | TAGTCGGGAGCTTCCTCTCC | 80 |
| *Lat2* | CTGCTCACCAGAGTCAGTGG | GCTGTAAGGCTGAGGGTGAG | 99 |
| *Pag1* | GCACAACTTCAAAGCTGGGAG | TCATCCTCCTTGGAGAAACGG | 106 |
| *Lst1/A* | GCACAACCAATGATTTCCTGCT | CGGCACAAGCAGATGAACAG | 91 |

**Supplementary table 4. Antibodies**

| **Antibody** | **Application** | **Dilution** | **Manufacturer** |
| --- | --- | --- | --- |
| Mouse anti-V5 tag | WB  IF | 1:2500  (0.4 μg mL^-1^)  1:1000  (1 μg mL^-1^) | Bio-Rad, California, USA |
| Mouse anti-Myc tag | WB    IF | 1:1000  (1 μg mL^-1^)  1:8000  (0.125 μg mL^-1^) | Cell Signalling Technology, Boston USA |
| Mouse anti-GM130 | IF | 1:1000  (0.25μg mL^-1^) | BD Bioscience, San Jose, USA |
| Rabbit anti-GAPDH | WB | 1:1000  (1 μg mL^-1^) | Cell Signalling Technology, Boston, USA |
| Goat HRP-linked anti-rabbit IgG | WB | 1:2500  (0.4 μg mL^-1^) | Cell Signalling Technology, Boston, USA |
| Goat HRP-linked anti-mouse IgG | WB | 1:2500  (0.4 μg mL^-1^) | Cell Signalling Technology, Boston, USA |
| Goat Alexa 568 anti-mouse IgG | IF | 1:1666  (1.2 μg mL^-1^) | Life technologies, Carlsbad, USA |
| Human Rhodamine anti-GAPDH | WB | 1:10000 | Bio-Rad, California, USA |
| Mouse anti-Tlr4 | WB | 1:1000  (1 μg mL^-1^) | Abcam, Cambridge, UK |
| Rabbit anti-Scimp^1^ | WB  IP | 1:300  1:200 | WEHI, Victoria, Australia |
| Mouse anti-phosphotyrosine (4G10) | WB | 1:1000 | Merck, Darmstadt, Germany |
| Rat anti-mouse Il-6  (Capture) | ELISA | 1:500  (1 μg mL^-1^) | BD Biosciences, California, USA |
| Rat biotinylated anti-mouse Il-6  (Detection) | ELISA | 1:500  (1 μg mL^-1^) | BD Biosciences, California, USA |
| Rat anti-mouse Il-12p40  (Capture) | ELISA | 1:250  (4 μg mL^-1^) | BD Biosciences, California, USA |
| Rat biotinylated anti-mouse Il-12p40 (Detection) | ELISA | 1:500  (2 μg mL^-1^) | BD Biosciences, California, USA |
| Rat anti-mouse Tnf  (Capture) | ELISA | 1:250 | BD Biosciences, California, USA |
| Rat biotinylated anti-mouse Tnf  (Detection) | ELISA | 1:250 | BD Biosciences, California, USA |

**References**

1. Luo L, Bokil NJ, Wall AA*, et al.* SCIMP is a transmembrane non-TIR TLR adaptor that promotes proinflammatory cytokine production from macrophages. *Nat Commun* 2017; **8**: 14133.
